# Modeling the Dynamic Properties of Multi-Layer Glass Fabric Sandwich Panels

**DOI:** 10.3390/polym16213074

**Published:** 2024-10-31

**Authors:** Arkadiusz Charuk, Izabela Irska, Paweł Dunaj

**Affiliations:** 1 Faculty of Mechanical Engineering and Mechatronics, West Pomeranian University of Technology in Szczecin, al. Piastów 19, 70-310 Szczecin, Poland; 2 Marine Ship Repair Yard “Gryfia” J.S.C., Brdowska 12 Str., 71-700 Szczecin, Poland

**Keywords:** sandwich panel, modal analysis, finite element method, glass fabric, sensitive analysis

## Abstract

Sandwich panels are key components of many lightweight structures. They are often subjected to time-varying loads, which can cause various types of vibrations that adversely affect the functionality of the structure. That is why it is of such importance to predict the dynamic properties of both the panels and the structures made of them at the design stage. This paper presents finite element modeling of the dynamic properties (i.e., natural frequencies, mode shapes, and frequency response functions) of sandwich panels made of glass fabric impregnated with phenolic resin. The model reproducing the details of the panel structure was built using two-dimensional, quadrilateral, isoparametric plane elements. Afterwards, the model was subjected to an updating procedure based on experimentally determined frequency response functions. As a result, the average relative error for natural frequencies achieved numerically was 5.0%. Finally, a cabinet model consisting of the analyzed panels was built and experimentally verified. The relative error between the numerically and experimentally obtained natural frequencies was on average 5.9%.

## 1. Introduction

Currently, a substitute for plywood is being sought as a material for building furniture for floating units. This substitute should be characterized by functional properties at a similar level to plywood while reducing the weight of the furniture [1]. The use of sandwich-structured composites can be considered as a promising material solution for such applications.

The sandwich-structured composites are designers’ favorite materials when high rigidity and low mass are required [2]. These composites consist of two thin, stiff outer layers called face sheets or skins and a lightweight but relatively thick middle layer called the core. The core material is usually of low stiffness, but by providing a gap between the skins, it gives the composite structure with high flexural stiffness at an overall low density [3]. Sandwich composites are characterized by high diversity in terms of materials used which include, among others: metal-faced sandwich composites [4], wood-based sandwich composites [5], z-pinned sandwich composites [6], or bio-inspired ones [7].

A group that is gaining popularity exceptionally quickly, due to, among other things, ease of production [8] and good mechanical properties [9], are glass fiber-based composites. Although much research has been conducted on the dynamic properties of sandwich panels [10], the rapid development of this type of structure seems to continually generate a demand for new research involving both experimental characterization and modeling methods.

Petrůet et al. [11] presented a study of the mechanical properties of samples from long fiber-reinforced composite structures. The possibilities and methods of measuring composite reinforced with carbon and glass fibers were described. Analytical models were introduced to describe the cross-sectional isotropic composite, including the mathematical relationships that allow the determination of unknown elastic constants. Finally, problems related to the creation of a numerical model of a composite fibrous structure were outlined and specified mechanical properties and the interaction between fibers and the matrix. The built numerical model reflects the concept of a coherent continuum, which consists of a surface geometry that corresponds to the sample being tested. Authors pay attention to the connection of fibers with the matrix because the joints form an interphase. This is influenced not only by the material parameters and boundary conditions but also by the proper selection of a chosen type of finite element mesh. The authors show that the type of finite element chosen determines the quality of the results obtained, and its choice must be dictated by the purpose of the study.

Sadighi et al. [12] performed experimental studies of three-dimensional woven glass-fiber sandwich composites and presented the results of finite element simulations to investigate the mechanical behavior of the panels. The tested sample was made of Parabeam 3D woven E-glass fabric and impregnated with Derakane 411-45 epoxy vinyl ester resin. Mechanical tests, such as the flatwise and edgewise compression test, shear test, and bending test, were performed by a Zwick testing machine with axial actuators. The numerical model consisted of two parts, fabric and resin, using two different methods of modelling. Element T3D was selected for the glass fabric. The 3D10 element type for resin was used. The embedded element technique was used to model the connections between fabric and resin. Predicted and experimental curves for stress strain, compressive strength, compressive modulus, shear strength, and shear modulus were in good agreement. Thinner panels exhibited higher contact rigidity and perforation load, while thicker panels exhibited higher energy absorption capacity.

Mirdehghan et al. [13] focused on the micromechanical-analytical model as a method for the prediction of the compressive strength of three-dimensional integrated woven sandwich composite panels. The composite sandwich panels consisted of two fabric surfaces that were bonded by a core. The core contained vertically pilled yarns that connected both two face sheets. The pile yarn connected the sheets in the warp direction (similar to the form “8” shape) and the weft direction (similar to the form “c” shape). The authors described in detail the geometry of the core pile as the most important step for developing a micromechanical model. The mechanical properties of fiber and resin used for the finite element model were provided by the manufacturer. The flatwise compression of the sample in the thickness direction was performed by the Instron tensile testing machine (model 5566). The presented nine comparisons of experimental and predicted load deflection show an acceptable agreement between both results. The average relative error between experimental and predicted strength was 6.2%, but the maximum relative error was 12%.

Selvaraj et al. [14] investigated the mechanical and dynamic properties of natural fiber-reinforced composite sandwich panels with multi-core layers. The structure featured jute fiber-reinforced polymer composite face sheets and cores made from natural rubber and cork. Using ABAQUS, the authors developed a three-dimensional finite element model to study how different ply stacking sequences affect the free vibrations of single-core and multi-core sandwich panels. The validation of the model was achieved through comparison with previously published results, demonstrating the model’s reliability.

In a related study, Prasad et al. [15] explored the free vibration and bending properties of hybrid jute composite beams. The authors developed finite element models based on the higher-order theory, and their results—covering natural frequencies, deflection, and stress—were in good agreement with the existing literature, further verifying the accuracy of their model.

Zheng et al. [16] focused on enhancing the damping performance of carbon laminates by integrating a novel viscoelastic layer within the structure. A dynamic analytical model was constructed to assess the damping properties of the co-cured sandwich composite structure based on geometric equations and constitutive relations. Using the Rayleigh–Ritz method, the authors derived the natural frequencies and loss factors. The strong alignment between the finite element simulations, theoretical derivations, and experimental modal tests validated both the theoretical framework and experimental design.

In the summary of the literature review, it should be stated that there are many works devoted to experimental studies and the modeling of static properties of glass fiber-based composites. However, not many works concern their dynamic properties, and considering the research background, i.e., seeking for an alternative material to produce furniture for vessels, dynamic properties are an important aspect, as one can observe many sources generating vibrations on floating units.

This paper presents a procedure for modeling the dynamic properties of a multi-layer glass fabric sandwich panel with particular emphasis on natural frequencies, mode shapes, and frequency response functions, as they are the primary determinants characterizing the structure in terms of dynamics and are frequently analyzed in the design process of mechanical structures (e.g., furniture for vessels).

As part of the research, a composite panel finite element model based on experimentally determined material and geometrical parameters was built. To increase the model mapping accuracy, the model updating procedure (preceded by a sensitivity analysis) was then performed based on experimentally determined frequency response functions. The modeling procedure and identified model parameters were then validated by predicting the dynamic properties of a composite panel with different dimensions. Finally, to prove the method’s practical utility, the model of complex structure was built and subjected to experimental verification.

The novelty of this paper lies in the modelling procedure considering the composite structure (shape and separate parameters for the core, reinforcement through-the-thickness, and facings) as well as the sensitivity analysis carried out on its basis provides a broader insight into how individual composite elements affect its dynamic properties and can be used for future optimization of similar structures. An additional distinguishing feature of the work is the use of developed models to predict the dynamic properties of a complex structure such as the presented cabinet.

The structure of this paper is as follows: In Section 2, the materials and methods are described. This section contains descriptions of research objects, experimental stands, and finite element models. In Section 3, the results of the finite element analysis and its experimental verification are presented. The discussion of the obtained results is presented in Section 4, while Section 5 encapsulates the conclusions, summarizing the most significant achievements of the work.

## 2. Materials and Methods

### 2.1. Research Object

**Figure 1:**
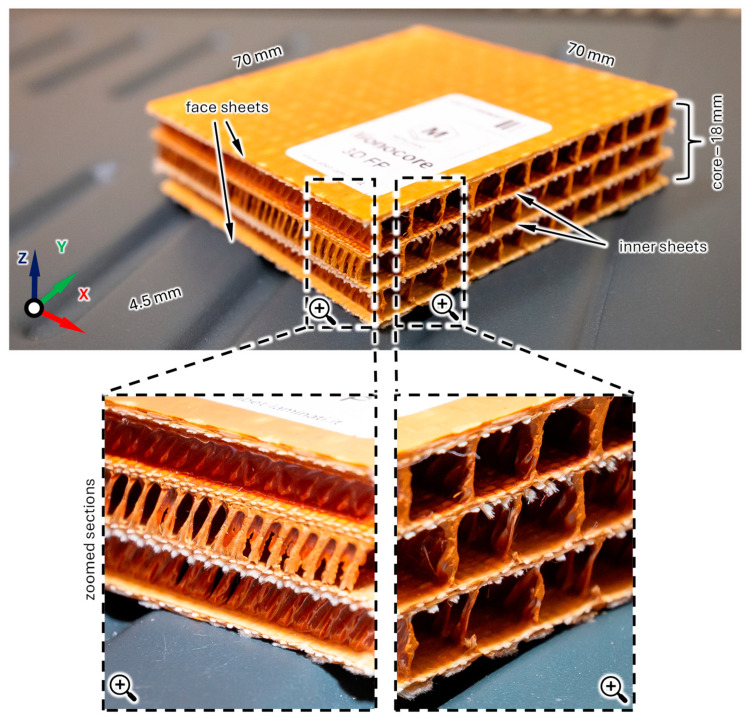
Structure of a multi-layer glass fabric sandwich panel—general view.

The research object is Abet Monocore 3D FP, a multi-layer glass fabric sandwich panel (Metalleido Components s.r.l., Borgo Fornari, Italy). The panel is made entirely of glass fiber impregnated with phenolic resin. It consists of two face sheets and two inner sheets interconnected by impregnated woven cross-links (reinforcement through the thickness) that form a series of hollow square tunnels. The general view of the panel structure is depicted in Figure 1.

In the present study, two composite panels differing in dimensions are used. The first panel has dimensions 630 × 565 × 18 mm and weighs 2.5 kg, and the second has dimensions 396 × 790 × 18 mm and weighs 2.2 kg.

### 2.2. Geometric Measurements

**Figure 2:**
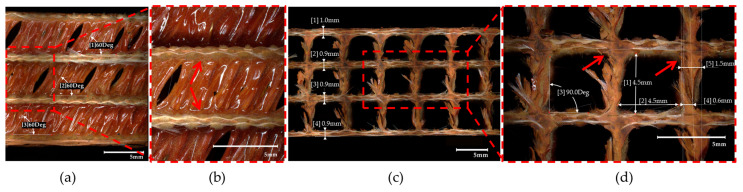
The cross-sectional optical images of the analyzed panel: parallel to the tunnel alignment (**a**,**b**), perpendicular to the tunnel alignment (**c**), and hollow tunnel geometry (zoomed view) (**d**).

The Monocore 3D FP manufacturing technology is patented, and the manufacturer does not provide information on the material properties or the detailed geometric structure. Therefore, to specify the key features defining the structure of the panel, it was examined employing microscopic imaging using a high-accuracy 4K digital microscope VHX-7000 series (Keyence, Osaka, Japan). Figure 2 gathers the magnified cross-sectional optical images of the analyzed panel. The thickness of individual layers and the dimensions of the hollow sections were measured multiple times, and the representative values are provided in the images.

With regard to the reinforcement through the thickness, it was established that it is non-continuous and laid at a 60-degree angle to the face sheets (Figure 2a). It was found that the individual sheets differ in thickness. The top sheet can be considered the thickest, with an average thickness of 1 mm, whilst the internal and bottom sheets are slightly thinner, each measuring ca. 0.9 mm (Figure 2c). The hollow tunnels are roughly rectangular, measuring 4.5 × 4.5 mm (Figure 2d). Furthermore, a close inspection of micrograms presented herein reveals a good connection between particular plies of woven glass fabric and spacer yarns (reinforcement through-the-thickness). This can be seen in both the images taken parallel and perpendicular to the tunnel alignment (Figure 2b,d respectively), particularly at the points marked with red arrows. Glass fiber yarns seem to be pulled out from the individual piles, forming a reinforcement along the *Z*-axis. Given the discontinuity of the *Z*-axis reinforcement and its geometry (arranged with a slope of 60°), it is obvious that some Z-reinforcement fibers appear to be torn when cut perpendicular to the tunnels.

### 2.3. Static Tests

**Figure 3:**
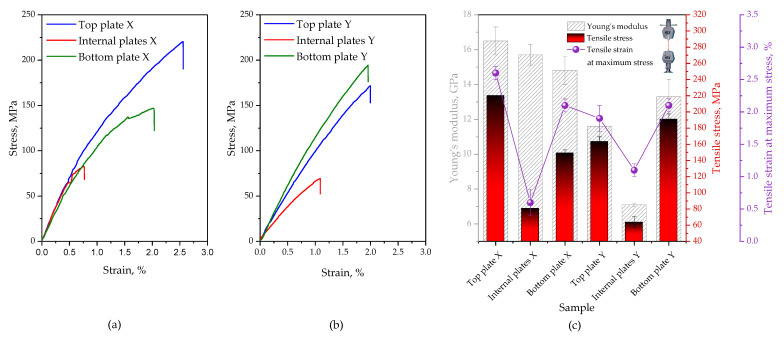
Representative stress-strain curves of top, internal, and bottom plates of the analyzed panel tested perpendicular (**a**) and parallel (**b**) to the z-reinforcement. Summary of tensile characterization (**c**).

To determine the material properties of the analyzed panel required for finite element modeling, static tests were performed. The tensile tests of polymeric composites were determined following the ISO 527-4 standard [17] (in compliance with test conditions for orthotropic fiber-reinforced plastic composites) and ISO 178 standard [18] (determination of flexural properties). Three types of samples taken from different parts of the analyzed panel were considered, specifically the top and bottom face sheet as well as the inner sheet. Rectangular specimens of 250 × 25 mm^2^ for stress-strain and 80 × 10 mm^2^ for flexural tests were examined along with the respective sheet thicknesses. Assuming that one is dealing with orthotropic material, each sheet was tested in two directions, i.e., spacer yarns oriented perpendicular and parallel to the sample’s length, further denoted as X and Y, respectively. An Autograph AG-X plus universal testing machine (Shimadzu, Tokyo, Japan) equipped with a 10 kN load cell, TRViewX non-contact type video extensometer, non-shift wedge grips with file-teeth grip faces for tensile tests, and 3-point bend fixture for flexural tests was employed. Tests were performed at room temperature at a crosshead speed of 2 mm/min. The tensile stress ($s_{tM}$) and tensile strain at maximum stress ($e_{tM}$), flexural stress ($s_{fM}$), and flexural strain at flexural strength ($e_{fM}$) were determined. Poisson’s ratio and the tensile and flexural elastic modulus (n,$E_{t}$ and $E_{f}$, respectively) were calculated from the initial linear slope of the stress-strain curve (from 0.05 to 0.25% strain). To obtain a reliable average value and standard deviation, at least seven samples were tested. Representative stress-strain curves are depicted in Figure 3.

**Figure 4:**
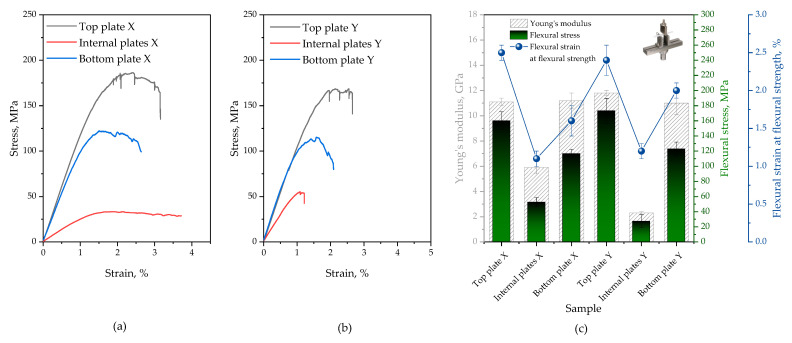
Representative stress-strain curves of top, internal, and bottom plates of the analyzed panel tested perpendicular (**a**) and parallel (**b**) to the z-reinforcement; summary of flexural characterization (**c**).

For illustrative purposes, stress-strain curves and the summary of the flexural characterization regarding Young’s modulus, flexural stress, and flexural strain at flexural strength are presented in Figure 4. The most important numerical values that summarize the static test results are given in Table 1.

### 2.4. Finite Element Model

The finite element model of the analyzed panel was built using a Midas NFX 2023 R1 preprocessor (Midas Information Technology Co., Ltd., Seongnam, Republic of Korea). A structured mesh for each component constructed from quadrilateral, four-node, isoparametric plane finite elements (CQUAD) was used. The utilized finite elements were characterized by linear shape functions and six degrees of freedom in each node (three translational and three rotational). Based on the preliminary analysis of the influence of the size of the applied finite elements on the obtained eigenvalue results, the size of the finite elements corresponding to the height of a single layer of through-the-thickness reinforcement was selected. The connection between individual components was carried out using the coincidence of nodes. The orthotropic material model MAT8 was used to describe the material properties of subsequent components. The structural damping model was used to describe the damping properties of the plates and walls according to which the damping matrix ***C*** can be expressed as:

(1) C=iηK

where K is the stiffness matrix, i is the imaginary unit, and η is the loss factor.

**Figure 5:**
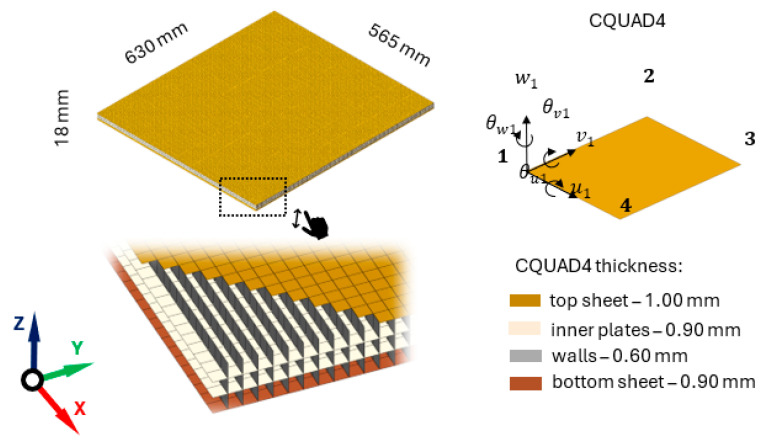
Finite element model of the analyzed panel.

The developed model has 99,666 finite elements and 347,472 degrees of freedom. To minimize the impact of the unmodeled environment on their dynamic properties (also at a later stage of experimental testing), the model was unconstrained. The finite element model of the analyzed panel is shown in Figure 5.

### 2.5. Dynamic Tests

To verify the developed models as well as to gather information on the damping present in the structure, an experimental modal analysis in the form of an impact test was performed. To approximate the free boundary conditions, the analyzed panels were subsequently suspended on nylon strings, and the place of their attachment was selected in such a way as to best match the nodes of the analyzed mode shapes.

The panel was excited using the PCB 086C01 modal hammer (PCB Piezotronics, Depew, NY, USA) in a direction perpendicular to the face sheet (*Z*-axis). Analyzing the coherence function obtained from preliminary studies, it was decided that the experimental modal model would be composed of two partial experiments performed with different hammer tips (soft and medium hard) in order to ensure the best possible excitation conditions. More precisely, the mode shapes in the range from 0 to 500 Hz were estimated based on the frequency response functions determined for the soft tip excitation, while those in the range from 500 to 1000 Hz were estimated based on the medium hard tip excitation.

**Figure 6:**
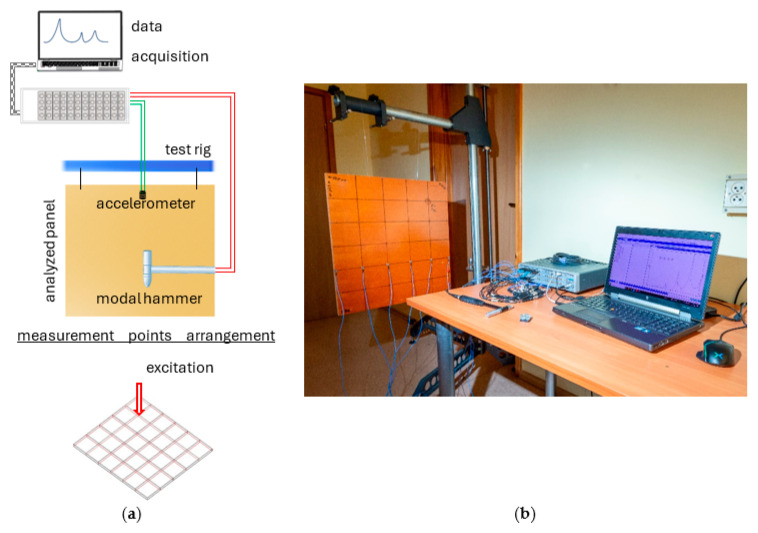
Modal analysis test stand with measurement points arrangement: schematic representation (**a**) and actual stand (**b**).

The responses of the analyzed panel were measured in 84 points (42 points each for top face and bottom face) using the PCB 356A01 three-axis piezoelectric accelerometer (PCB Piezotronics, Depew, NY, USA). Data acquisition was performed using Scadas Mobile Vibco and Testlab 2019.1 software (Siemens AG, Munich, Germany). The estimation of frequency response functions was performed with the use of an H_1_ estimator. The remaining parameters of signal acquisition were as follows: sampling rate 4096 Hz; frequency resolution 0.5 Hz; number of averages 10. The test stand with the measurement point arrangement is depicted in Figure 6.

As a result of the impact test conducted, 84 frequency response functions were determined on the basis of which, using the Polymax algorithm [19,20], the parameters of the modal model of the analyzed panel were estimated. The obtained modal model was validated using the MAC criterion, eliminating interdependent vectors in the mode shape (the limit value of 10% was assumed) [21]. Moreover, based on the experimental results, it was possible to identify the loss factor value, which in the analyzed case was η=0.0075.

## 3. Results

### 3.1. Experimental Verification

**Figure 7:**
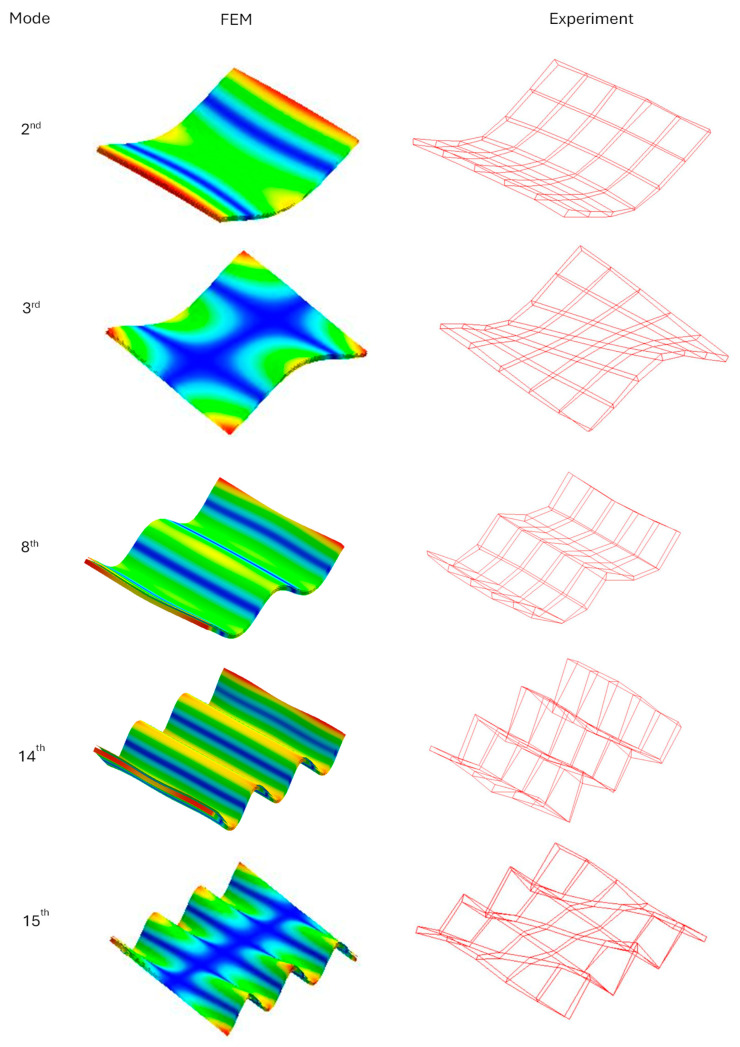
Comparison of selected mode shapes determined numerically and experimentally.

**Figure 8:**
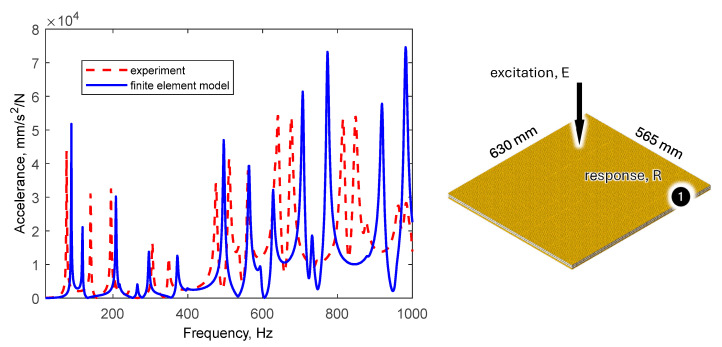
Comparison of selected frequency response functions determined numerically and experimentally.

The comparison of the values of natural frequencies for the analyzed panel obtained numerically (using SOL103 solver) and experimentally supplemented with the relative error value is shown in Table 2. A comparison of selected modes is shown in Figure 7. A comparison of the accelerance functions determined numerically (using SOL108 solver) and experimentally is shown in Figure 8.

Analyzing the obtained results, it can be seen that in the analyzed frequency range, all sixteen mode shapes were correctly identified. However, after analyzing the obtained natural frequencies, it can be seen that the maximum error is 16.3%, and on average 10.6%. With this in mind, it can be concluded that the values of material properties obtained on the basis of static tests are not sufficient. Therefore, it was decided to include a model updating algorithm in the procedure.

### 3.2. Model Sensitivity Analysis

**Figure 9:**
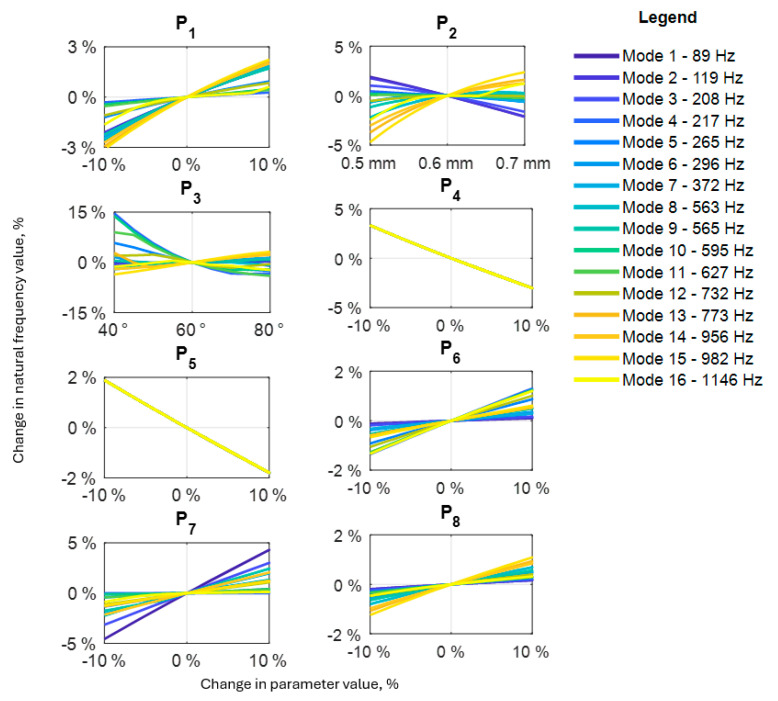
Sensitivity analysis results for analyzed panel.

To select appropriate decision variables for the model updating as well as to have insight into how changing individual parameters affects the dynamic properties of the model, a sensitivity analysis was performed by changing the values of selected model parameters in the range from 90% to 110% of their nominal value while observing how these changes affect the natural frequencies. The parameters adopted for the sensitivity analysis are shown in Table 3, while the results are depicted in Figure 9.

Analyzing the obtained results, it can be seen that changes in through-the-thickness reinforcement parameters, such as thickness (*P*_2_) and angle (*P*_3_), have the greatest impact on the change in natural frequencies. The nature of these changes is in general non-linear. Observed non-linearity increases as the natural frequency values increase. Similar dependencies can be observed for the parameter *P*_1_, with the difference that their influence on the change of natural frequencies is much smaller.

The remaining parameters that have a significant impact on the values of natural frequencies are those related to the stiffness of the structure: $P_{7}$ (shear modulus of the sheets) and its mass $P_{4}$ and $P_{5}$ (material density of sheet’s and through-the-thickness reinforcement’s, respectively). Here, the nature of the changes is mainly linear, which can be explained by a direct analogy to the stiffness-to-mass ratio, which reflects the natural frequency for a system with one degree of freedom.

### 3.3. Model Updating

For the model updating process, parameters presented in Section 3.2, Table 3, were adopted. As the accelerance amplitudes indicated in Figure 7 show satisfactory agreement, the damping ratio was not updated.

The process of identifying the model parameters is reduced to the task of minimizing the objective function, formulated as follows [22]:

(2) $$Q=\left[{\left[{\ddot{y}}^{exp}-{\ddot{y}}^{fem}\right]}^{T}\left[{\ddot{y}}^{exp}-{\ddot{y}}^{fem}\right]\right]$$

where ${\ddot{y}}^{exp}$ is the experimentally determined accelerance function, and ${\ddot{y}}^{fem}$ is the accelerance function determined based on the finite element model.

The model identified parameters are shown in Table 4, while a comparison of the natural frequencies is shown in Table 5.

**Figure 10:**
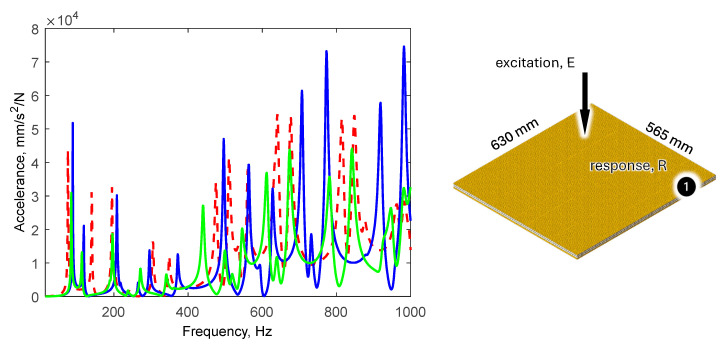
Comparison of selected frequency response functions determined numerically (before and after model updating) and experimentally.

Figure 10 shows a comparison of the frequency response functions for the updated model.

Updating the model resulted in a significant improvement in natural frequency agreement. The average relative error decreased from 10.6% to 5.0%. In addition, the frequency response functions improved.

### 3.4. Validation of the Modeling Method

**Figure 11:**
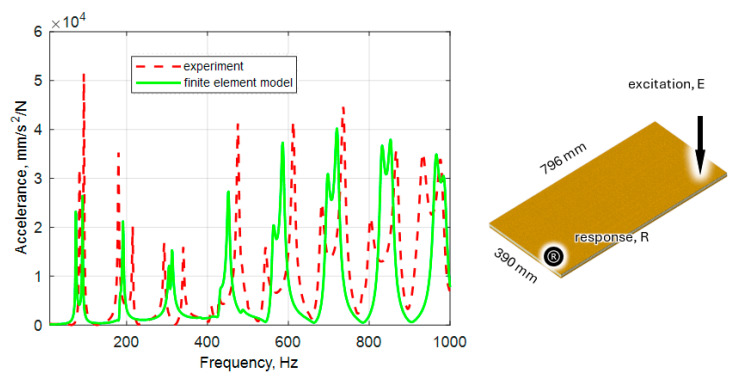
Comparison of selected frequency response functions determined numerically and experimentally for 390 × 796 × 18 mm panel.

The validation of the modeling procedure was carried out using different panels measuring 396 × 790 × 18 mm. The panel was modeled according to the procedure presented and then experimentally verified. It should be noted that the material parameters used came from the model updating process presented in Section 3.3 (Table 4). The obtained results in the form of natural frequencies are presented in Table 6, while the comparison of frequency response functions is depicted in Figure 11.

### 3.5. Cabinet Model

In the previous subsections, only unconstrained models of multi-layer glass fabric sandwich panels were considered, obtaining—thanks to the proposed modelling method—a high agreement of calculations with experimental results. In reality, however, certain structures are built using those panels. Therefore, in order to prove the utility of the method a model of a cabinet was built, which was then subjected to experimental verification.

The cabinet main body has the dimensions of 400 × 600 × 800 mm. It consists of the following elements made of 18 mm thick multi-layer glass fabric sandwich panels: two sides, a bottom shelf, and two rails. These elements are connected to each other by a dedicated connector for structural panels Ixconnect 32/20 (Häfele SE & Co KG, Nagold, Germany). In addition, the formats are glued along the entire length of their contact. The cabinet door with a push lock handle is also made of a multi-layer glass fabric sandwich panel and has dimensions of 776 × 396 mm. It is attached to the main body with two hinges. The main body is topped with a panel measuring 420 × 630 mm. The cabinet does not have a back plate as it is dedicated to vessels, in which furniture is attached to the wall using dedicated connectors.

**Figure 12:**
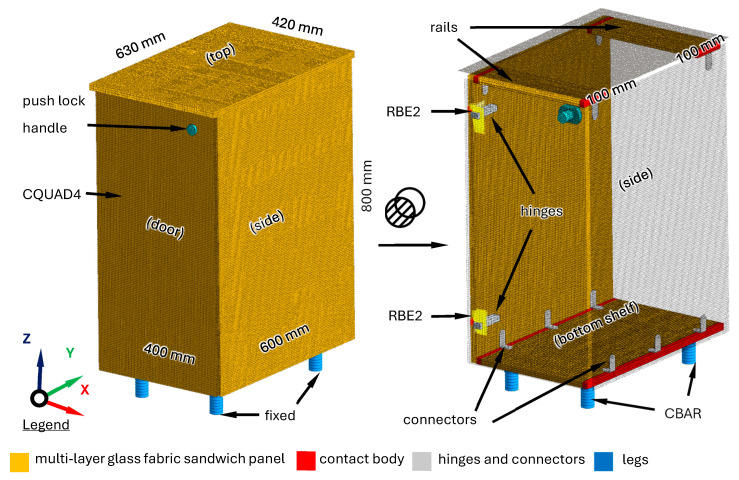
A finite element model of cabinet.

The finite element model of the cabinet was built using the finite element model of a multi-layer glass fabric sandwich panel established in Section 2.4, with material parameters identified in Section 3.3. and listed in Table 4. The connections of individual elements of the plywood cabinet were based on the third body contact model [23]. In the case of connecting the door with the main body, in order to reliably reproduce the connection nature (especially the area where it takes place), additional rigid finite elements (RBE2) were used [24]. The cabinet legs were modeled using a method presented in [25]. In total, the cabinet model consisted of 512,219 elements and had 1,795,290 degrees of freedom. Next, the model was constrained at the legs, where the actual cabinet touches the floor. The cabinet structure together with the finite element model established is depicted in Figure 12.

**Figure 13:**
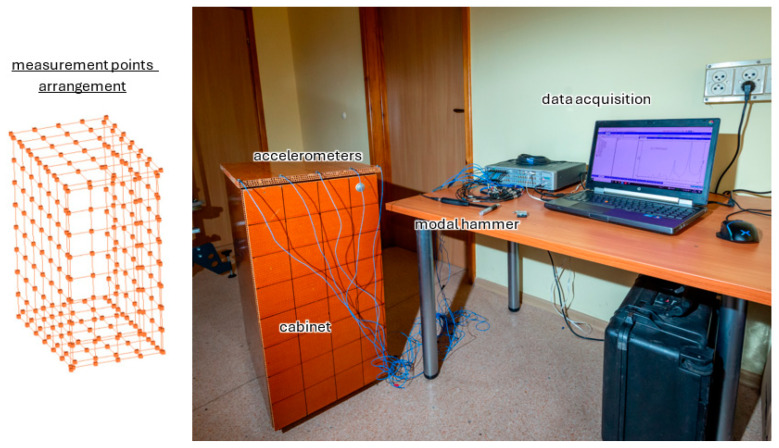
Modal analysis test stand—cabinet testing.

To verify the developed model, an experimental modal analysis was carried out in the form of an impact test using the test stand presented in Section 2.5. A photo of the test stand is shown in Figure 13.

**Figure 14:**
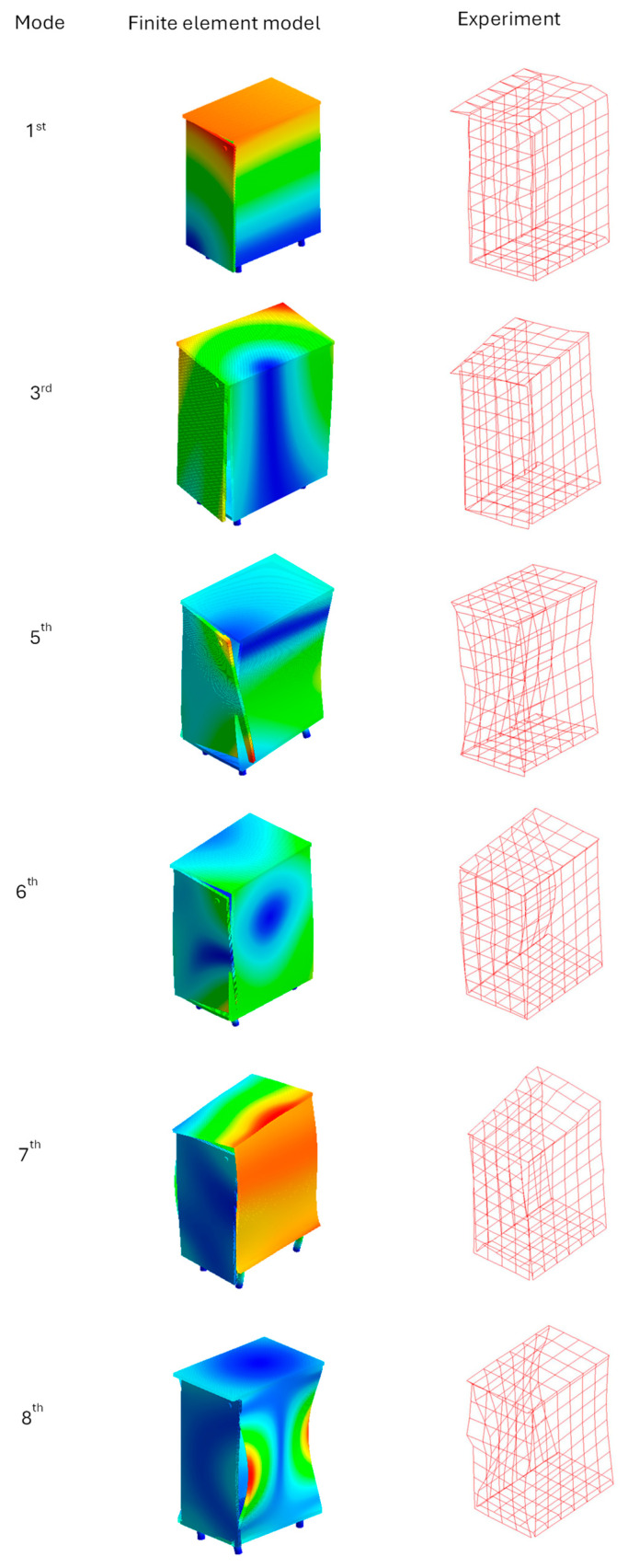
Comparison of cabinet mode shapes determined numerically and experimentally.

**Figure 15:**
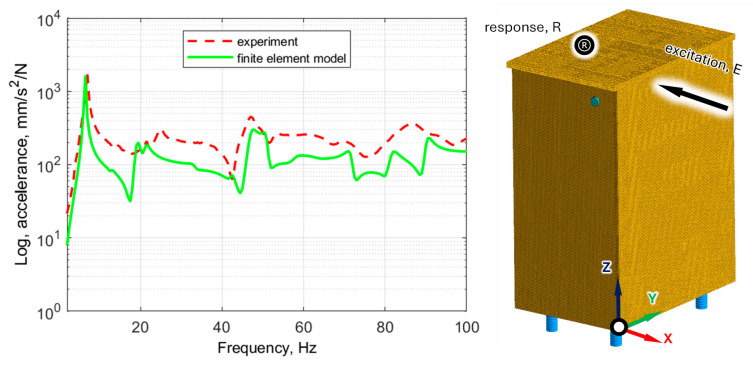
Comparison of frequency response functions for cabinet determined numerically and experimentally.

Table 7 presents the experimental verification of natural frequencies, Figure 14 presents selected mode shapes comparison, and Figure 15 presents the experimental verification for exemplary frequency response functions.

The alignment of the acceleration function determined for the FEM model and on the basis of the experiment is shown in Figure 15.

## 4. Discussion

This discussion herein is divided into two main parts: the first one covers the results of static tests, and the second one concerns the dynamic part (both model and experiment).

Referring to the static tests, the tensile and flexural properties of the multi-layer glass fabric sandwich panel were investigated. As pointed out earlier, the through-the-thickness reinforcement is discontinuous and should be considered the weakest point of the composite panel under investigation. One can expect that the thin and non-continuous reinforcement throughout the thickness undergoes shear failure at low loadings. According to the manufacturer’s specifications, the capacity of the structure to withstand loads under compression perpendicular to the panel face attains 1.28 MPa, whilst tensile strength measured perpendicular to the panel face does not exceed 1.15 MPa. Thus, the current study is focused on the mechanical performance of individual sandwich panels—i.e., on the sections with potentially higher resistance to mechanical loads. It has been well established in the literature that fiber-reinforced composites, especially unidirectional ply composites, are highly orthotropic, whilst the woven fabric ply typically offers more balanced mechanical properties [26].

To better understand the behavior of the multilayer structure in question, the top, internal, and bottom sheets in two directions, perpendicular and parallel to the through-the-thickness reinforcement, were tested with respect to tensile and flexural properties. By comparing the stress-strain curves from the outer (top and bottom) and inner sheets of the panel, it is apparent that the former are stiff in both directions and are able to support greater tensile loads, whilst the latter show markedly deteriorated tensile properties. The tensile properties of each specimen were found dependent on both plate type and load direction. A Young’s modulus of 16.5 ± 0.8 GPa, tensile strength of 220.2 ± 0.2 MPa, and elongation at a maximum strength of 2.6 ± 0.1% achieved with a face sheet-top plate tested perpendicular to the through-the-thickness reinforcement is the highest among the investigated samples. Slightly inferior results were obtained for samples collected from the bottom sheet and samples taken from the face sheets of the panel with reinforcement parallel to the test direction. Although the internal sheets with *z*-axis reinforcement perpendicular to the tensile direction retained a large value of modulus of elasticity ($E_{t}\;$= 15.7 ± 0.6 GPa), one can notice that the stress at break and elongation at maximum stress decreased substantially ($s_{tM}$ = 80.7 ± 3.7 MPa, $e_{tM}\;$= 0.6 ± 0.25%). The same sheets stretched parallel fractured under even lower stress values ($s_{tM}\;$= 63.6 ± 6.9 MPa) at an elongation of ~1% while displaying significantly reduced stiffness ($E_{t}$ reduced by ~65% compared to the plates tested perpendicular to the reinforcement). It was found that all panel sheets exhibit roughly similar values of Poisson’s ratio (from 0.24 for internal panels up to 0.25 for most of the face sheets). The top and bottom plates of the panel analyzed herein exhibit comparable flexural rigidity in both directions ($E_{f}$ varies around 11.0 GPa). As far as face sheets are concerned, the bottom ones were found to exhibit reduced flexural strength and lower elongation at maximum flexural stress compared to the top ones (a 30% drop in $s_{fM}$ and an 18–35% drop in $e_{fM}$ was noted depending on the load direction). Irrespective of load direction, the internal plates demonstrated significantly reduced mechanical properties (reduced $E_{t}$, $s_{fM}$, $e_{fM}$) when compared to the outer plates of the panel.

On the basis of the two different experimental approaches (tensile and flexural tests), it was affirmed that different sheets significantly differ in terms of mechanical performance. This difference is particularly evident when comparing face and inner sheets. A likely explanation for this behavior could be the manufacturing process and construction of the sandwich panel. As expected, due to the orthotropic nature of the material analyzed the test direction affected the obtained results. Also noteworthy is the fact that load direction has a more pronounced effect on the tensile properties of the laminates, whereas the flexural performance remains roughly unaffected.

Referring to the dynamic part, it can be stated that the presented methodology for modeling the dynamic properties of a multi-layer glass fabric sandwich panel is able to effectively predict its dynamic properties, which has been confirmed by experimental verification. However, the analysis presented in this paper indicates that building a finite element model based on the values of material parameters derived from static tests gives significant discrepancies in the values of the natural frequencies. Hence, to achieve high accuracy, it seems necessary to use a model updating approach, which allowed for a decrease in the average relative error from 10.6 % to 5.0 % in the case of the analyzed panel. Additionally, an improvement in the accuracy of mapping the accelerance was achieved. Analyzing the identified values of model parameters, it can be noticed that most of them are ±11% variability (except for the parameters of the through-the-thickness reinforcement $P_{6},P_{8}$, the variability of which is in the range of ±130%). Due to significant differences, these values may raise some concerns, although considering the characteristics of the material itself and the production process, these differences seem to be acceptable. However, to fully support this statement, the validation of both the modeling method and identified parameters was conducted. It consisted of predicting the dynamic properties of a composite panel of different sizes than the one used in the model updating process. As a result for a 390 × 796 × 18 mm panel, the maximum error for the natural frequencies was 22.1%, on average 6.3%, which can be considered satisfactory. Additionally, all mode shapes in the analyzed frequency range were reproduced by the model, and satisfactory agreement of frequency response functions was achieved.

The research summary presents an example of the application of the developed methodology to predict the dynamic properties of a complex system such as a cabinet intended for vessels. As a result, the maximum error for the natural frequency values was 27.3% and on average 5.9%. Moreover, the satisfactory agreement of mode shapes and frequency response functions was achieved. The presented application example can be considered as an additional validation stage, although it is not direct as it considers other elements in the model such as hinges and connections between panels and legs.

## 5. Conclusions

Modeling the dynamic properties of multi-layer glass fabric composites is a difficult task due to the non-uniformity of the material, resulting from, among other things, the production process. In an attempt to contribute to the subject, this paper presents the finite element modeling of the dynamic properties of a multi-layer glass fabric sandwich panel. The dynamic properties analyzed were natural frequencies, mode shapes, and accelerance. The experimental verification of the model proved its effectiveness in terms of predicting listed dynamic properties. The method’s practical utility was demonstrated by building a cabinet model and verifying it experimentally. As a result, satisfactory agreement in terms of natural frequencies, mode shapes, and frequency response functions was achieved.

The main limitation of this study seems to be a poor representation of the through-the-thickness reinforcement in the model. This is manifested by significant differences between the parameter values before and after model tuning. This may indicate that the initial parameters adopted were inaccurate. However, it is particularly difficult to examine an isolated reinforcement sample and therefore to provide reliable material parameters characterizing it.

Another shortcoming of this study is the problem of accurately reproducing mode shapes for lower frequencies. Importantly, this problem persists even after tuning the model. Due to the similarity of low mode shapes to static defection shapes, the results of static tests performed for panels could provide deeper insight into this problem. Therefore, future work should focus on trying to solve these problems.

## Figures and Tables

**Table 1:** Elastic constants together with tensile and flexural properties of particular sheets of analyzed panel.

Sample	E_t_, GPa	ν	σ_tM_, MPa	ε_tM_, %	E_f_, GPa	σ_fM_, MPa	ε_fM_,%
perpendicular to the sample’s length, X							
face sheet (top)	16.5 ± 0.8	0.25 ± 0.01	220.2 ± 0.2	2.6 ± 0.1	11.1 ± 0.3	160.1 ± 11.9	2.5 ± 0.1
inner plates	15.7 ± 0.6	0.24 ± 0.01	80.7 ± 3.7	0.6 ± 0.2	5.9 ± 0.5	52.6 ± 5.9	1.1 ± 0.1
face sheet (bottom)	14.8 ± 0.8	0.25 ± 0.01	149.3 ± 3.8	2.1 ± 0.1	11.2 ± 0.6	116.8 ± 5.6	1.6 ± 0.2
parallel to the sample’s length, Y							
face sheet (top)	11.6 ± 0.6	0.24 ± 0.01	163.4 ± 5.7	1.9 ± 0.2	11.8 ± 0.2	173.3 ± 16.4	2.4 ± 0.2
inner plates	7.1 ± 0.1	0.24 ± 0.01	63.6 ± 6.9	1.1 ± 0.1	2.3 ± 0.1	27.3 ± 8.7	1.2 ± 0.1
face sheet (bottom)	13.3 ± 1.0	0.25 ± 0.01	191.1 ± 6.4	2.1 ± 0.1	11.0 ± 0.9	122.8 ± 9.1	2.0 ± 0.1

**Table 2:** Experimental verification of natural frequencies for model of analyzed panel (initial parameters).

Mode Shape	Tip	Experiment, Hz	Finite Element Model, Hz	Relative Error, %
1	soft	77	89	15.6
2		141	119	15.6
3		195	208	6.7
4		204	217	6.4
5		244	265	8.6
6		306	296	3.3
7		349	372	6.6
8		476	496	4.2
9	medium hard	511	563	10.2
10		537	595	10.8
11		561	627	11.8
12		643	707	10.0
13		678	773	14.0
14		816	919	12.6
15		846	982	16.1
16		985	1146	16.3
		**Average**		**10.6**

**Table 3:** Parameters adopted for the sensitivity analysis.

Designation	Description
*P*_1_	Sheets thickness
*P*_2_	Through-the-thickness reinforcement thickness
*P*_3_	Angle of the through-the-thickness reinforcement fibers
*P*_4_	Density of the sheet’s material
*P*_5_	Density of the through-the-thickness reinforcement’s material
*P*_6_	Modulus of elasticity of the through-the-thickness reinforcement, simultaneous change of $E_{XX}$, $E_{YY}$
*P*_7_	Shear modulus of the sheets, simultaneous change of $G_{XY}$, $G_{YZ}$, $G_{ZX}$
*P*_8_	Shear modulus of the through-the-thickness reinforcement, simultaneous change of $G_{XY}$, $G_{YZ}$, $G_{ZX}$

**Table 4:** Material data for analyzed panel.

Parameter		Unit	Initial Value	Identified Value	Relative Difference, %
$P_{1}$	Sheets thickness	mm	0.90	0.85	5.6
$P_{2}$	Through-the-thickness reinforcement thickness	mm	0.60	0.65	8.3
$P_{3}$	Angle of the through-the-thickness reinforcement fibers	degrees	60	60	0
$P_{4}$	Density of the sheet’s material	kg/m^3^	1800	2000	11.1
$P_{5}$	Density of the through-the-thickness reinforcement’s material	kg/m^3^	1800	1700	5.6
$P_{6}$	Modulus of elasticity of the through-the-thickness reinforcement, $E_{XX}$	MPa	100,000	40,000	60.0
	Modulus of elasticity of the through-the-thickness reinforcement, $E_{YY}$	MPa	14,000	5600	60.0
$P_{7}$	Shear modulus of the sheets, $G_{XY}$	MPa	5685	5685	0
	Shear modulus of the sheets, $G_{YZ}$	MPa	5314	5314	0
	Shear modulus of the sheets, $G_{ZX}$	MPa	5314	5314	0
$P_{8}$	Shear modulus of the through-the-thickness reinforcement, $G_{XY}$	MPa	27,700	4575	83.5
	Shear modulus of the through-the-thickness reinforcement, $G_{YZ}$	MPa	1400	3218	129.9
	Shear modulus of the through-the-thickness reinforcement, $G_{ZX}$	MPa	1400	3218	129.9

**Table 5:** Experimental verification of natural frequencies for updated model of analyzed panel.

Mode Shape	Experiment, Hz	Finite Element Model (Initial Parameters), Hz	Relative Error, %	Finite Element Model (Identified Parameters), Hz	Relative Error, %
1	77	89	15.6	85	10.4
2	141	119	15.6	114	19.1
3	195	208	6.7	196	0.5
4	204	217	6.4	190	6.9
5	244	265	8.6	238	2.5
6	306	296	3.3	271	11.4
7	349	372	6.6	341	2.3
8	476	496	4.2	441	7.4
9	511	563	10.2	499	2.3
10	537	595	10.8	518	3.5
11	561	627	11.8	545	2.9
12	643	707	10.0	612	4.8
13	678	773	14.0	673	0.7
14	816	919	12.6	782	4.2
15	846	982	16.1	841	0.6
16	985	1146	16.3	980	0.5
		**Average**	**10.6**	**Average**	**5.0**

**Table 6:** Experimental verification of the natural frequencies of 396 × 790 × 18 mm panel.

Mode Shape	Experiment, Hz	Finite Element Model (Identified Parameters), Hz	Relative Error, %
1	83	91	9.6
2	95	74	22.1
3	180	191	6.1
4	215	183	14.9
5	294	305	3.7
6	341	312	8.5
7	408	385	5.6
8	429	408	4.9
9	476	452	5.0
10	545	562	3.1
11	614	586	4.6
12	684	696	1.8
13	739	721	2.4
14	802	830	3.5
15	869	855	1.6
16	930	964	3.7
		**Average**	**6.3**

**Table 7:** Experimental verification of mode shapes for cabinet.

Mode Shape	Experiment, Hz	Finite Element Model, Hz	Relative Error, %
1	7	7	0
2	11	14	27.3
3	19	20	5.3
4	34	33	2.9
5	42	43	2.4
6	57	56	1.8
7	72	69	4.2
8	90	93	3.3
		**Average**	**5.9**

## Data Availability

The original contributions presented in the study are included in the article, further inquiries can be directed to the corresponding author.
